# The Complexity of Fungal β-Glucan in Health and Disease: Effects on the Mononuclear Phagocyte System

**DOI:** 10.3389/fimmu.2018.00673

**Published:** 2018-04-16

**Authors:** Giorgio Camilli, Guillaume Tabouret, Jessica Quintin

**Affiliations:** ^1^Immunology of Fungal Infections, Department of Mycology, Institut Pasteur, Paris, France; ^2^Université de Toulouse, INRA, INP, ENVT, IHAP, Toulouse, France

**Keywords:** fungal, β-glucan, mononuclear, phagocyte, health, disease

## Abstract

β-glucan, the most abundant fungal cell wall polysaccharide, has gained much attention from the scientific community in the last few decades for its fascinating but not yet fully understood immunobiology. Study of this molecule has been motivated by its importance as a pathogen-associated molecular pattern upon fungal infection as well as by its promising clinical utility as biological response modifier for the treatment of cancer and infectious diseases. Its immune effect is attributed to the ability to bind to different receptors expressed on the cell surface of phagocytic and cytotoxic innate immune cells, including monocytes, macrophages, neutrophils, and natural killer cells. The characteristics of the immune responses generated depend on the cell types and receptors involved. Size and biochemical composition of β-glucans isolated from different sources affect their immunomodulatory properties. The variety of studies using crude extracts of fungal cell wall rather than purified β-glucans renders data difficult to interpret. A better understanding of the mechanisms of purified fungal β-glucan recognition, downstream signaling pathways, and subsequent immune regulation activated, is, therefore, essential not only to develop new antifungal therapy but also to evaluate β-glucan as a putative anti-infective and antitumor mediator. Here, we briefly review the complexity of interactions between fungal β-glucans and mononuclear phagocytes during fungal infections. Furthermore, we discuss and present available studies suggesting how different fungal β-glucans exhibit antitumor and antimicrobial activities by modulating the biologic responses of mononuclear phagocytes, which make them potential candidates as therapeutic agents.

## Introduction

β-glucans are naturally occurring glucose polymers that are present in abundance in plants, bacteria, and fungi. For centuries, traditional Chinese medicine uses fungi for healing and currently, interests have focused on polysaccharides that are a crucial component of fungi cell walls (1). Within the multitude of polysaccharides present, β-glucans are a key reason fungi are used in cosmetics, as food additives, or as medicinal purposes (2); they have also shown beneficial effects in the outcome of various diseases (3). β-glucans, share a common structure consisting of a backbone of β(1,3)-linked β-d-glucopyranosyl units. However, they can strongly differ by their length and branching structure. Fungal β-glucans, that represent the most abundant polysaccharides found in the cell wall of fungi, are mainly characterized by the presence of β(1,6)-linked branches coming off of the β(1,3) backbone. The structural diversity also depends on the fungal source (4). For example, β-glucans of mushrooms have short β(1,6)-linked branches whereas those of yeast have β(1,6)-side branches with additional β(1,3) regions (5). Of note, these structural differences may influence the immunogenic properties of β-glucans and many studies have suggested that a higher degree of structural complexity is associated with enhanced β-glucans-induced antimicrobial and anticancer activities (6).

β-glucans as the most abundant fungal cell wall polysaccharide in human fungal pathogens, is also a key pathogen-associated molecular pattern (PAMP) that is detected upon fungal infection to trigger the host immune responses in vertebrates and invertebrates (7). Different membrane-bound immune receptors can recognize β-glucans and receptor binding is also dependent on the structure and nature of the β-glucan (8). In fact, the cellular responses induced by mushroom or yeast β-glucans depend on their specific interactions with several cell surface receptors (PRRs), as scavenger receptors, lactosylceramide (LacCer), complement receptor 3 (CR3; CD11b/CD18), and dectin-1. Although the binding of β-glucan to LacCer (9) or scavenger receptors (10) on the cell surface of leukocytes has been described, the biological mechanisms that can result from these interactions and the effects on the immune responses are still lacking and need further investigations.

Complement receptor 3 is a heterodimeric transmembrane glycoprotein consisting of CD11b noncovalently associated with CD18 and mainly expressed on neutrophils, monocytes, and natural killer (NK) cells. Besides its first reported role in triggering phagocytosis and degranulation in response to yeast iC3b-opsonized yeast (11, 12), CR3 also has a β-glucans-specific binding site that map to a lectin-site C-terminal of the I-domain of the CD11b (13). β-glucan binding to the lectin site of the CR3 on phagocytes and NK cells primes the receptor to enhance the cytotoxicity against iC3b-opsonized target cells, including tumors, that are otherwise resistant to CR3-dependent cytotoxicity (14, 15). Recently, much attention has been focused on dectin-1, predominantly expressed on the cell surface of monocyte, macrophages, neutrophils, and dendritic cells (DCs), which has emerged as the primary β-glucan receptor (16–18). Interestingly, however, there has been some controversy about the relative importance of the various β-glucan receptors, and their activity seems to be tightly dependent on the cell context. In fact, while neutrophil modulation by β-glucan is predominantly CR3-dependent, dectin-1 is the most important β-glucan receptor on macrophages (19, 20).

Dectin-1 is a type II transmembrane protein characterized by the presence of an extracellular C-type lectin-like domain, a stalk, a transmembrane region, and an intracellular region with an immunoreceptor tyrosine-based activation motif (ITAM)-like motif containing a single tyrosine residue (21, 22). Binding of β-glucan to dectin-1 induces the phosphorylation of the hemi-ITAM, phosphorylation of Syk, and activation of the CARD9/Bcl10/MALT-1 (caspase recruitment domain/B cell CLL-lymphoma 10/mucosa associated lymphoid tissue lymphoma translocation gene 1) signaling complex, which in turn leads to the activation of the downstream signaling pathway (23). Moreover, Dectin-1 can also induce signaling *via* Raf-1 in a Syk-independent fashion as well as through the PI3K/Akt pathway (24, 25). As a consequence of these signaling activations, dectin-1 triggers phagocytosis, ROS generation, microbial killing, and cytokine production. Of note, β-glucan/dectin-1 interaction *per se* is sufficient to induce the phagocytosis and production of ROS. In contrast, the production of inflammatory cytokines seems to be dependent on the cooperation between dectin-1 and toll-like receptors (TLRs) (26–28).

The high structural variability, low purity, and the ability to bind to different receptors are probably the main limitations of current β-glucan research. Differences in the molecular weight, degree of branching, triple helical conformation, and solubility may affect the binding affinity of β-glucan to each receptor leading to activation of multiple and variable signaling pathways. A systematic investigation of the receptor binding, the signaling pathway, and the activated immune responses induced by pure β-glucans with known structure is, therefore, needed to better understand the fungal pathogenesis but also to effectively apply the use of the β-glucan for the treatment of cancer and infectious diseases.

The objectives of this review are to present the different functions triggered by mononuclear phagocytes upon an encounter with β-glucans covering the mechanisms of recognition and the importance of β-glucans structural diversity, as well as the interaction of the β-glucan with the host during an infection. We will also explore the effects of fungal β-glucan treatment on cancer and infectious diseases and finally discussed the recently described innate immune memory of monocytes associated with β-glucans training.

## β-Glucan-Mediated Interactions of Fungal Pathogens with Mononuclear Phagocytes

A diverse group of fungi is known to be pathogenic for humans, including *Candida* spp., *Cryptococcus neoformans, Aspergillus fumigatus*, and *Histoplasma capsulatum*. Fungal pathogenic organisms can cause diseases varying from mild infections of the skin and cutaneous tissues to severe invasive infections when tissue homeostasis is compromised. Of note, the prevalence of fungal infections registered a dramatic increase during the last decades due to a variety of factors, including AIDS epidemic and the use of immunosuppressive treatments in cancer and transplanted patients (29). An intact immune system is, therefore, essential to control fungal infections, and mononuclear phagocytes play a pivotal role in host defense against fungal invasion (30).

Since the discovery of specific PRRs for β-glucan on the cell surface of phagocytes (i.e., CR3 and dectin-1), this abundant cell wall polysaccharide has drawn increasingly more attention as a key PAMP in the host immune recognition of pathogenic fungi. However, some fungal species have developed surface structures to evade such immune control mechanism. For example, *H. capsulatum* can mask the recognition of the immunogenic β-(1,3)-glucan by phagocytic receptors through a less immunogenic outer layer of α-(1,3)-glucan (31). Similarly, *C. neoformans* hides their surface immunogenic molecules, including β-(1,3)-glucan, behind a polysaccharide capsule thus inhibiting phagocytosis and cytokine production by macrophages (32). Pathogenicity of dimorphic fungi, such as the yeast *Candida albicans*, is known to be linked to their capacity to adapt and switch back and forth between the filamentous and yeast growth. Interestingly, β-glucan, which is normally masked by the mannan layer of the yeast cell wall, becomes exposed at the budding scar, but not during the filamentous growth. Thus, the process of the budding growth has been suggested as the target for dectin-1 recognition by macrophages (33). Moreover, β-glucan structure and activity differ between *C. albicans* yeast and hyphae (34). Interestingly, dectin-1, in contrast to other PRRs, discriminates between soluble and particulate β-glucans (35). Phagocytosis and cytokine production by macrophages are only induced when dectin-1 is bound to particulate β-glucan or live fungi through the formation of a “phagocytic synapse” and the exclusion of regulatory phosphatases (35). This process represents a unique mechanism to discriminate PAMPs associated with a microbial surface.

Recognition of the fungal PAMPs by phagocytes and the subsequent engulfment of the pathogen *via* phagocytosis represent the first steps to control fungal infections [as reviewed in Ref. (36)]. A series of signaling events are required to mediate phagocytosis when dectin-1 binds to the β-glucan-rich fungal cell wall. As mentioned above, clustering of dectin-1 with the concomitant exclusion of CD148 and CD45 phosphatases are essential steps to induce hemi-ITAM phosphorylation and recruitment of Syk kinase (35). However, the requirement of Syk in the phagocytic process is controversial, and its role is probably cell type dependent. In fact, the inactivation of Syk does not impair the phagocytosis of β-glucan-containing particles in genetically or pharmacologically inactivated macrophages (28, 37). On the opposite, Syk knockdown DCs are unable to phagocytose zymosan particles (38). Moreover, dectin-1 translocation to β-glucan-containing phagosomes and the subsequent Syk activation allow the acidification and maturation of *C. albicans* and β-glucan-containing phagosomes in macrophages (39). β-glucan binding to dectin-1 is, therefore, not only important to trigger phagocytosis but also to allow the lysosomal fusion and acidification of the phagocytic compartment. It is likely that other molecules can mediate a dectin-1-dependent phagocytosis through related or independent pathways besides the hemi-ITAM-mediated Syk activation. For instance, Vav1 and PI3K are required for dectin-1/β-glucan-dependent phagocytosis in microglial cells (25). Similarly, PI3K and the RhoGTPase inhibitors significantly reduce the internalization of zymosan particles in RAW2643.7 cells expressing dectin-1 (28).

Recognition of β-glucan by Dectin-1 triggers autophagy through an LC3-associated phagocytosis and directs LC3 recruitment to phagosomes containing fungi (40). Importantly, this mechanism regulates the subsequent immunological response. The association of LC3 to the β-glucan-containing phagosomes increases MHC II recruitment to phagosomes and presentation of fungal-derived antigens to CD4 T cells by DCs (41).

Besides the phagocytosis and the acidification of macrophage phagosome, the production of oxidative molecules is another important mechanism in killing fungal pathogens. β-glucan/Dectin-1 interaction followed by Syk activation is crucial for the generation of ROS in mononuclear phagocytes (37, 42, 43). However, whether dectin-1-mediated oxidative burst is a universal key mechanism in controlling fungal killing remains to be elucidated. As such, dectin-1 activation and ROS production in macrophages are essential for killing of *Pneumocystis carinii* but not *C. albicans*, suggesting that the mechanism is only required for immune responses to some fungal infection (44). Interestingly, form and structure of β-glucan impact on the type of oxidative burst triggered in human monocytes. More precisely, particulate and phagocytizable β-glucan activates the NADPH-dependent reaction *via* dectin-1 while immobilized and non-phagocytizable one triggers the reaction in a CR3-dependent fashion (45). Moreover, ROS production can affect the antifungal immune responses by regulating a multiplicity of other mechanisms, such as autophagy and inflammasome activation (46).

Although it is well known that purified β-glucan is able to activate phagocytosis and production of ROS, its ability to induce cytokine production is still debated. Production of inflammatory mediators by fungi or crude β-glucan preparation (i.e., zymosan) is mediated by a collaboration between dectin-1 and TLRs. For example, production of TNF-α and IL-12 in macrophages and DCs upon stimulation with β-glucan-containing particles is due to a synergism between dectin-1 and TLR2 (27). When the zymosan is hot alkali-treated to remove its TLR-stimulating properties, the particles are still internalized and induce dectin-1 activation and ROS production. On the opposite, the depleted zymosan fails to induce cytokine production, suggesting that dectin-1/β-glucan signaling is not enough *per se* to trigger a robust release of inflammatory cytokines (27). Similarly, the production of TNF-α in response to zymosan or live fungi requires activation of dectin-1 as well as TLR2 and Myd88 (26). In addition, TNF-α production is strongly decreased in dectin-1-knockout macrophages treated with *C. albicans* or zymosan (43). Therefore, high inflammatory signature of mononuclear phagocytes induced by β-glucan seems to be linked to a multiple receptor activation, but dectin-1 remains a crucial component in this network. Intriguingly, β-glucan binding to dectin-1 only, strongly induces the release of inflammatory molecules by mononuclear phagocytes when phagocytosis is impaired by actin polymerization inhibitors (47–49). Therefore, phagocytosis of β-glucan is also involved in the modulation of dectin-1 signaling and the weak mediated inflammatory response (47–49).

Many efforts have been recently made to examine the role of fungi and β-glucan in inflammasome activation and subsequent production of IL-1β. NLRP3 inflammasome plays a crucial role in regulating antifungal immune responses and host survival. Mice with impaired NLRP3 present increased fungal burden and decreased survival to *C. albicans, A. fumigatus*, or *C. neoformans* infections (50–53). β-glucan and fungi trigger the induction and processing of IL-1β in mouse DCs *via* dectin-1 and through the activation of a non-canonical caspase-8 inflammasome (54). The inflammasome is a multiprotein complex that regulates the processing and release of IL-1β and IL-18 but also triggers pyroptosis, a form of cell death of the infected cell that is distinct from classical apoptosis or necrosis and represents an efficient effector mechanism to protect the host from infection (55). *C. albicans* causes macrophage cell death by pyroptosis (56) and mutant of *C. albicans* that is defective in triggering pyroptosis has reduced β-glucan exposure in hyphae (57). However, to date, there are no convincing evidences that β-glucan itself can trigger pyroptosis directly.

## Effects of Fungal β-Glucan Treatment on Infectious Diseases and Cancer: Involvement of the Monocyte–Macrophage Axis

β-glucans are the key reason fungi are used in pharmacology and thought to positively impact cancer and infection evolution (3). *In vivo*, intramuscular administration of PGG-glucan, a highly purified soluble β-glucan isolated from *Saccharomyces cerevisiae*, results in an overall reduction in mortality and increase in absolute circulating numbers monocyte count in rats after a challenge with antibiotic-resistant *Staphylococcus aureus* (58). Monocytes isolated from untreated and β-glucan-treated mice show a different magnitude of cytokine response when stimulated *ex vivo* with either endotoxins or enterotoxins. Monocytes isolated from β-glucan-treated mice release a lower amount of pro-inflammatory cytokines involved in the pathogenesis of sepsis (i.e., TNF-α and IL-6) upon toxic stimulation, compared to cells isolated from control mice, suggesting a mechanism by which β-glucan treatment may reduce the host mortality during septicemia (59). Moreover, protection of glucan-treated mice from *Escherichia coli*-induced experimental peritonitis and bacteremia is due, in part, to an enhanced macrophage phagocytic function induced by the glucan (60). Lentinan, a (1,6)-branched (1,3)-β-glucan isolated from Japanese mushroom *Lentinus edodes* reduces *Mycobacterium tuberculosis* infection in mice and rats infected intraperitoneally and intranasally, respectively. Mouse peritoneal or rat alveolar macrophages show an increased acid phosphatase activity, free radicals production, and killing activity against *M. tuberculosis* (61, 62). In addition, *in vitro* stimulation of murine macrophages with Lentinan selectively attenuate AIM2 and non-canonical inflammasome activation. Accordingly, mice treated with Lentinan show a significant decrease in peritoneal IL-1β secretion after *Listeria monocytogenes* (AIM2 inflammasome trigger)-induced peritonitis (63). Lentinan administration also alleviates endotoxemic lethality of LPS-treated mice by inhibition of non-canonical inflammasome activation (63).

β-glucan has also been reported to have multiple antitumor properties and its effect may depend on the modulation of macrophage activity. The antitumor activity of GRN, a (1,6)-branched (1,3)-β-glucan obtained from mycelia of *Grifola frondosa*, is reduced when macrophage function are impaired with carrageean, suggesting a key role of macrophages in the antitumor-mediated mechanism (64). Peritoneal macrophages isolated from intraperitoneally lentinan-treated mice have a higher *in vitro* antitumor cytotoxic activity against murine or human target cells (65). Oral administration of *L. edodes* and *G. frondosa* counteract the inhibition of the chemotactic activity of macrophages induced by the carcinogen BBN (N-butyl-N-butanolnitrosoamine) (66). Blockage and inhibition in mice of dectin-1 expression on macrophages with mAbs, decrease the antitumor activity of SPG, a (1,6)-branched (1,3)-β-glucan from *S. commune* (67). Moreover, intravenous administration in mice of β-glucan isolated from *S. cerevisiae* strain reduces the colon 26-M3.1 carcinoma cell growth and increases the survival time of the tumor-bearing mice. These effects are associated with a higher production of pro-inflammatory cytokines and tumoricidal activity of peritoneal macrophages as well as an increased NK cell cytotoxicity (68). Orally administered β-glucan can enhance the tumoricidal activity of phagocytes toward iC3b-opsonized cancer cells. Schematically, the orally administered particulate yeast β-glucan is internalized by gastrointestinal macrophages and shuttles to the bone marrow where the glucan is degraded and released as a smaller size β-glucan fragments that are taken up by granulocytes *via* the CR3 receptor. The granulocytes with β-glucan-primed CR3 then kill iC3b/mAbs-coated tumor cells (69). Importantly, oral administration of β-glucan isolated either from mushrooms or yeast has the capacity to phenotypically convert the immunosuppressive M2 or tumor-associated macrophages into inflammatory M1 macrophages. In addition, the M2-to-M1 switch induced by β-glucan treatment leads to a reduced tumor burden (70, 71).

β-glucans also have potent hematopoietic activities by enhancing the production of hematopoietic factors, bone marrow recovery, as well as stem cell homing and engraftment. The hematopoiesis-stimulating properties of β-glucans are summarized in detail in the review by Hofer and Pospisil (72). Mice injected intraperitoneally with yeast β-glucan present macrophages with an altered morphology, increase in phosphatase activity as well as increased NO and superoxide production. These effects are especially observed when the animals are treated with β-glucan with a higher level of structural complexity in terms of molecular weight and degree of (1,6)-linkages (73). In addition, polysaccharides purified from the mycelium of *Ganoderma lucidum* (GL-PS), a medical mushroom commonly used in China, can impact immune cell proliferation and DCs maturation (74). GL-PS induces a proliferative response in human leukemia cell lines but also facilitates the maturation of DCs derived from THP1 monocytic leukemia cell line (75).

While animal studies seem promising, evidences for a human clinical application of the β-glucan are currently limited and does not fully support their recommendation. Most of the available clinical trials come from Eastern Countries and focused on the potential application of mushrooms in cancer therapy. A prospective clinical trial in patients with advanced breast cancer shows that administration of 10 mg capsules of soluble β-glucan from *S. cerevisiae* induces the proliferation and activation of peripheral blood monocytes with no clinical side effects (76). However, whether this can be clinically beneficial remains undisclosed. By contrast, the antimicrobial effect of β-glucan has been poorly investigated by clinical trials and results remain controversial. A phase I and II trials show that treatment with PGG-glucan reduces infection rates in high-risk surgery patients. However, while PGG-glucan administration lead to a reduction of serious infections and death, an increased incidence of adverse events is observed in patients receiving β-glucan treatment and phase III trial was terminated (77–79).

## β-Glucan Imprinting of Mononuclear Immune Cells

As described above, direct administration of fungal β-glucans may positively impact the outcome of a number of infectious diseases. Interestingly, past few years of research have enlightened a protective phenomenon triggered by the pre-administration of β-glucans, a mechanism mediated by monocytes and coined trained immunity (80). The observation that fungi could trigger a protective innate immune memory was first made upon the inoculation of non-germinating attenuated strains of the opportunistic human fungal pathogen *C. albicans*. Inoculation with this strain not only protects the mice against a virulent *C. albicans* but also against bacteria (81). The protection is independent of T lymphocytes, as observed in athymic mice (82), but dependent on macrophages (81) and pro-inflammatory cytokines (83). Macrophages are highly plastic cells and the innate immune system presents some adaptive properties (84, 85). The protection mediated is not restrained to avirulent fungal strains. Mice defective in adaptive T and B lymphocytes can be protected against re-infection with *C. albicans* in a monocyte-dependent manner and using a virulent strain of fungi (86). These recent works shed light on the mechanisms behind the innate immune protection mediated. Within *C. albicans*, the β-glucan cell wall component of the yeast induces a functional reprogramming of monocytes leading to enhanced inflammatory responses *in vivo* in mice and *ex vivo* in humans (86). The β-glucan receptor dectin-1, as well as to a lesser extent the CR3 receptor, are mediating the signal and the non-canonical Raf-1 pathway, but not Syk pathway, are key components in the heightened immune status triggered in monocytes. Whole-genome transcriptional and epigenetic analyses have clearly demonstrated that in the process of β-glucans-induced training, many inflammatory genes are downregulated and others are not modified or even upregulated (87). β-glucans imprint the innate immune memory in monocytes through stable changes in histone methylation and acetylation, of promoters and enhancers (86, 87). More specifically, initial activation of gene transcription by the first β-glucans encounter is accompanied by the acquisition of specific chromatin marks, which are for some maintained even after the elimination of the stimulus. This enhanced epigenetic status of the mononuclear phagocytes, illustrated for example by the persistence of H3K4me1, characterizing “latent enhancers,” results in a stronger response to secondary stimuli upon a non-specific (non-fungal-related) secondary challenge. Pathway analysis of the different cluster of genes identified in the whole-genome transcriptional and epigenetic analyses highlight important immunological (cAMP-PKA activation) and metabolic (aerobic glycolysis) pathways (87, 88). These pathways play crucial roles in the induction and maintenance of trained immunity (87, 88). As such, β-glucans trained monocytes present a shift from oxidative phosphorylation toward glycolysis through an Akt/mTOR/HIF-1α-dependent pathway, a phenomenon reminiscent of the Warburg effect in cancer (88). Whether and how this shift influences epigenetic processes in trained immunity needs to be further investigated. However, glycolysis, glutaminolysis, and the cholesterol synthesis pathway are imperative for the induction of trained immunity by β-glucan in monocytes (89). Actually, fumarate accumulation through glutaminolysis integrates immune and metabolic circuits to induce monocyte epigenetic reprogramming by inhibiting KDM5 histone demethylases and fumarate itself induced an epigenetic program that mimics β-glucan-induced trained immunity (89).

Studies in patients and healthy volunteers have also helped understanding some of the crucial inflammatory effects required in trained immunity induced by β-glucan on monocytes. The immunological networks activated in trained monocytes depend on STAT1 activation, and defects in trained immunity have been reported in patients with chronic mucocutaneous candidiasis due to *STAT1* mutations (90). Finally, individuals with autophagy defects are unable to mount a full and potent trained immunity (91).

To our knowledge, only one trial has investigated the potential of β-glucan training effect in human circulating monocytes function (92). Oral β-glucan is inexpensive and well-tolerated and, therefore, thought to potentially represent a promising immunostimulatory compound for human use. In the randomized open-label intervention pilot-study, 15 healthy male volunteers absorbed a daily dose of 1,000 mg of β-glucan at once for 7 days (92). However, β-glucan is barely detectable in serum of volunteers at all time-points and neither cytokine production nor microbicidal activity of leukocytes are affected by orally administered β-glucan. The present study does not support the use of oral β-glucan to enhance innate immune responses in humans but does not preclude the use of a higher dosage of β-glucan to reach detectable level in the blood (92).

Regarding trained immunity in monocytes, it is important to consider the life span of these cells. Monocytes are cells with a short half-life in circulation, with studies suggesting it to be up to 1 day (93). Considering a long-lasting effect of the innate immune memory might, however, still be a valid hypothesis as orally administered β-glucans are taken up by macrophages and transported to some hematopoietic niches (69). Moreover, β-glucan administration to mice-induces expansion of hematopeitic progenitors in the bone marrow which is associated with cell metabolism and results in a beneficial response to chemotherapy-induced myelosuppression or secondary LPS challenge (94). Rather than a direct β-glucan contact with progenitors, modulation of hematopoietic progenitors is mediated by the immune mediators environment (IL-1, GM-CSF) (94).

## Conclusion

**Figure 1:**
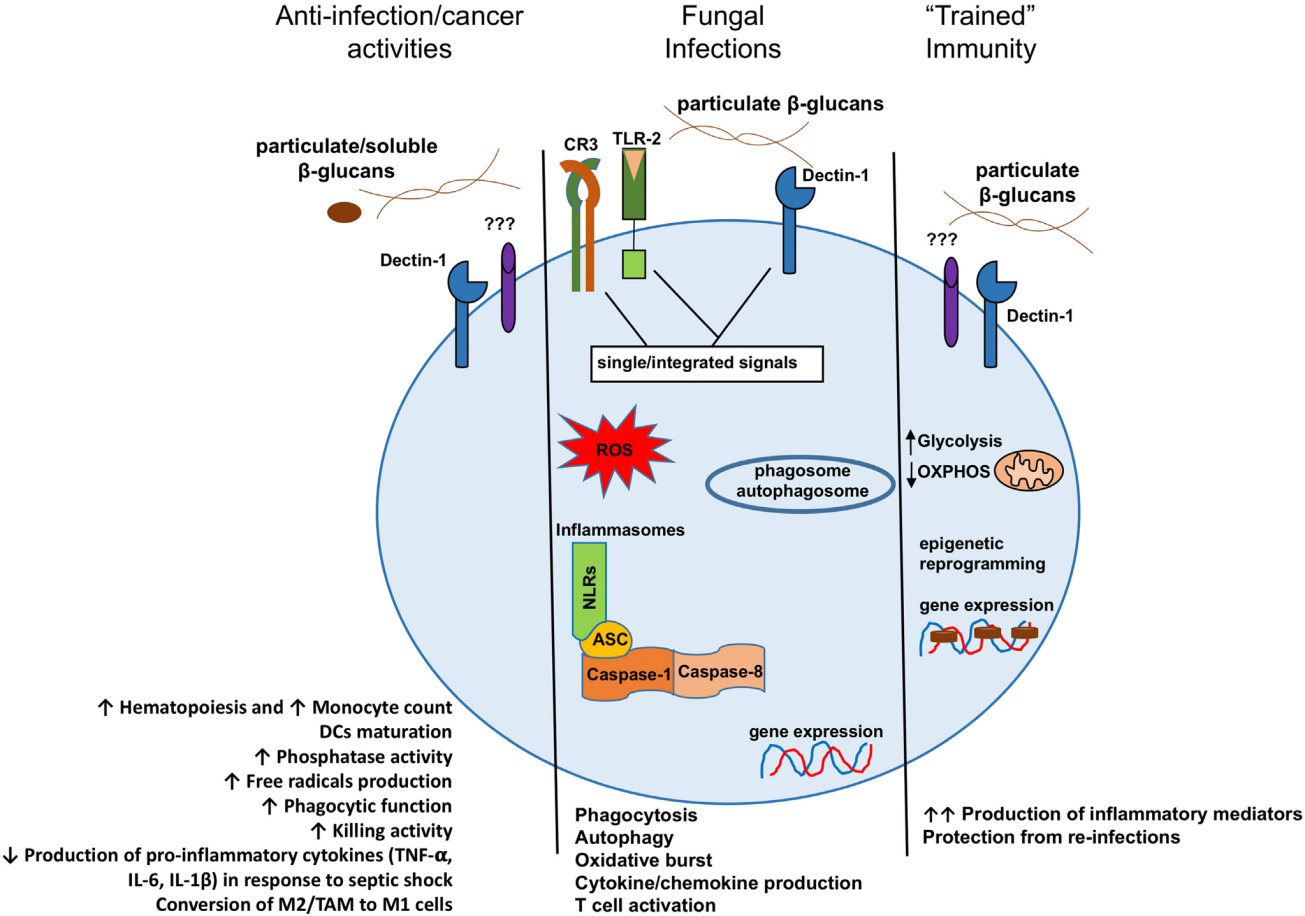
Model presenting the different consequences of β-glucan recognition by mononuclear phagocytes in the context of antitumoral activities, fungal infection recognitions, or trained immunity. DCs, dendritic cells; M2, alternative macrophages; TAM, tumor-associated macrophages; M1, classical macrophages.

Several experimental evidences have demonstrated a crucial role for β-glucan in the host–pathogen interaction during infections. Moreover, considerable efforts have been made to understand the cellular and molecular mechanisms of action of β-glucan in fungal pathogenesis as well as how it promotes a phagocytic-mediated immune response. Similarly, administration of fungal β-glucan is well known to stimulate the immune system and boost resistance to various infectious diseases and cancers, highlighting the multifaceted role of this molecule (Figure 1). However, although many *in vivo* studies have shown a beneficial effect of the β-glucans isolated from different sources, a comprehensive investigation of the mechanism of action is still lacking. In addition, the absence of detailed methodology on experimentation, β-glucan molecules source and purity reached render interpretation of the various results very complex. As such, discrepancies observed in the different studies are mainly related to the choice of purified components being used. In addition, unfortunately only few human studies are available and most of them have not been followed up with success. Hence, the possibility for clinical application of β-glucan should be considered with caution and will require further investigation. Future studies need to deeply characterize how β-glucans with different structure and molecular weight interact with each receptor and which specific signaling pathways are triggered. Moreover, providing details on the procedure and composition of the carbohydrate molecule under investigation remains crucial. An understanding should be made in the near future to use a common standardized β-glucan molecule with described biochemical properties. With such a common control, we might endeavor a rational use of this promising molecule in the future as an adjuvant or therapeutic agent.

## Author Contributions

GC, GT, and JQ jointly wrote the manuscript and approved it for publication.

## Conflict of Interest Statement

The authors declare that the research was conducted in the absence of any commercial or financial relationships that could be construed as a potential conflict of interest.
